# Radiation-induced lung injury in the era of combination anticancer therapy: a narrative review

**DOI:** 10.3389/fimmu.2026.1926641

**Published:** 2026-08-27

**Authors:** Xiuqiong Chen, Yi Duan, He Xiao, Zhengbo Wang, Xiao Yang, Chuan Chen, Mengxia Li

**Affiliations:** Cancer Center of Daping Hospital, Army Medical University, Chongqing, China

**Keywords:** combination therapy, pulmonary toxicity attribution, radiation-induced lung injury, systematic therapy, thoracic malignancies

## Abstract

Radiation-induced lung injury (RILI) remains a major dose-limiting toxicity of thoracic radiotherapy. In the era of combination anticancer therapy, its recognition and management have become more difficult. Systemic treatments can cause pneumonitis or interstitial lung disease on their own, but they can also change how radiation injury evolves. By sustaining inflammation or delaying repair of the irradiated lung, these agents may convert a typical radiation-related injury into a more complex pulmonary event. For this reason, post-treatment lung injury should not be interpreted solely by radiation dose or systemic therapy exposure. A RILI-centered approach may provide a clinically useful framework, allowing pulmonary events to be viewed as radiation-dominant RILI, interaction-modified RILI, or non-radiation mimics. In this narrative review, we summarize the pathological continuum of RILI, discuss how systemic therapies and treatment sequence influence its clinical expression, and outline a practical framework for diagnosis and management in combined-treatment settings. We also present a toxicity grade–based, attribution-informed management approach, ranging from observation and corticosteroid treatment to multidisciplinary care and selective immunosuppressive escalation for severe or steroid-refractory events. Antifibrotic strategies are considered separately for progressive fibrotic injury, alongside individualized decisions regarding systemic therapy interruption or rechallenge. This perspective may help clinicians balance pulmonary safety with the therapeutic benefits of modern combined-modality cancer treatment.

## 1 Introduction

Thoracic radiotherapy is a cornerstone of multidisciplinary treatment for lung cancer, esophageal cancer, and other thoracic malignancies, playing a critical role in improving local control and prolonging survival. However, RILI remains one of the major barriers to further optimizing the therapeutic benefit of thoracic radiotherapy (1). As one of the most important dose-limiting toxicities in thoracic irradiation, RILI not only compromises treatment tolerance and quality of life, but in severe cases may also lead to treatment interruption, delays in systemic therapy, and even life threatening outcomes (2). With the expanding integration of radiotherapy and systemic anticancer therapies, pulmonary toxicity has evolved from a predominantly dosimetric concern into a complex clinical entity shaped by multiple interacting factors (3).

In combined-modality treatment, lung injury is difficult to classify because systemic therapy can both mimic and modify RILI. Chemotherapy, targeted therapy, and immunotherapy may cause pulmonary adverse events independently (4), and these events often resemble RILI in symptoms, imaging, and timing (5). Yet the increased risk after combined treatment is not always a simple additive effect. Systemic therapy may also alter the course of radiation injury by increasing the vulnerability of irradiated lung tissue, sustaining inflammation, or delaying repair, thereby making RILI more severe or less confined to the expected radiation field (6, 7). Our understanding of these interactions, however, remains incomplete, and the pulmonary toxicity profiles associated with different drug classes and treatment sequences are markedly heterogeneous.

Accordingly, how systemic anticancer therapies influence the risk of lung injury after thoracic radiotherapy has emerged as an important question warranting renewed scrutiny. This review focuses on how systemic anticancer therapy and treatment sequence reshape the risk, presentation, and attribution of RILI after thoracic radiotherapy. We summarize representative evidence from chemotherapy, targeted therapy, antibody-drug conjugates, anti-angiogenic therapy, and immunotherapy, and propose a RILI-centered framework for interpreting pulmonary toxicity in combined-treatment settings.

## 2 Search strategy and evidence selection

PubMed, MEDLINE and Web of Science Core Collection were searched from database inception through 28 February 2026. The principal search terms were combined as follows: (“radiation-induced lung injury,” “radiation pneumonitis,” “radiation-induced pneumonitis,” “radiation-induced pulmonary fibrosis,” “radiation fibrosis,” “radiation recall,” “radiation recall pneumonitis,” and “radiation-induced organizing pneumonia”) and (“thoracic radiotherapy,” “thoracic irradiation,” “chest radiotherapy,” “chest irradiation,” “lung irradiation,” “mediastinal irradiation,” “stereotactic body radiotherapy,” “SBRT,” “SABR,” and “chemoradiotherapy”) and (“systemic anticancer therapy,” “chemotherapy,” “platinum,” “taxane,” “gemcitabine,” “pemetrexed,” “targeted therapy,” “tyrosine kinase inhibitor,” “EGFR-TKI,” “ALK inhibitor,” “antiangiogenic therapy,” “VEGF/VEGFR inhibitor,” “antibody–drug conjugate,” “immunotherapy,” “immune checkpoint inhibitor,” “PD-1 inhibitor,” “PD-L1 inhibitor,” and “CTLA-4 inhibitor”). Terms within each group were combined with OR, and the three groups were combined with AND. Reference lists of relevant reviews and guidelines were also screened. Eligible evidence comprised clinical trials, cohort studies, meta-analyses, guidelines or consensus statements, and lung-specific preclinical studies of radiation–systemic therapy interactions. Case reports were retained only for rare events, whereas duplicate or irrelevant reports and studies were excluded. Records were screened first by title and abstract, then by full text. Evidence selection prioritized guidelines, prospective studies, and large cohorts. Given the narrative design, no formal risk-of-bias assessment or quantitative synthesis was performed.

## 3 Conceptualizing RILI in the era of combination anticancer therapy

### 3.1 RILI as a dynamic injury–repair continuum

**Figure 1:**
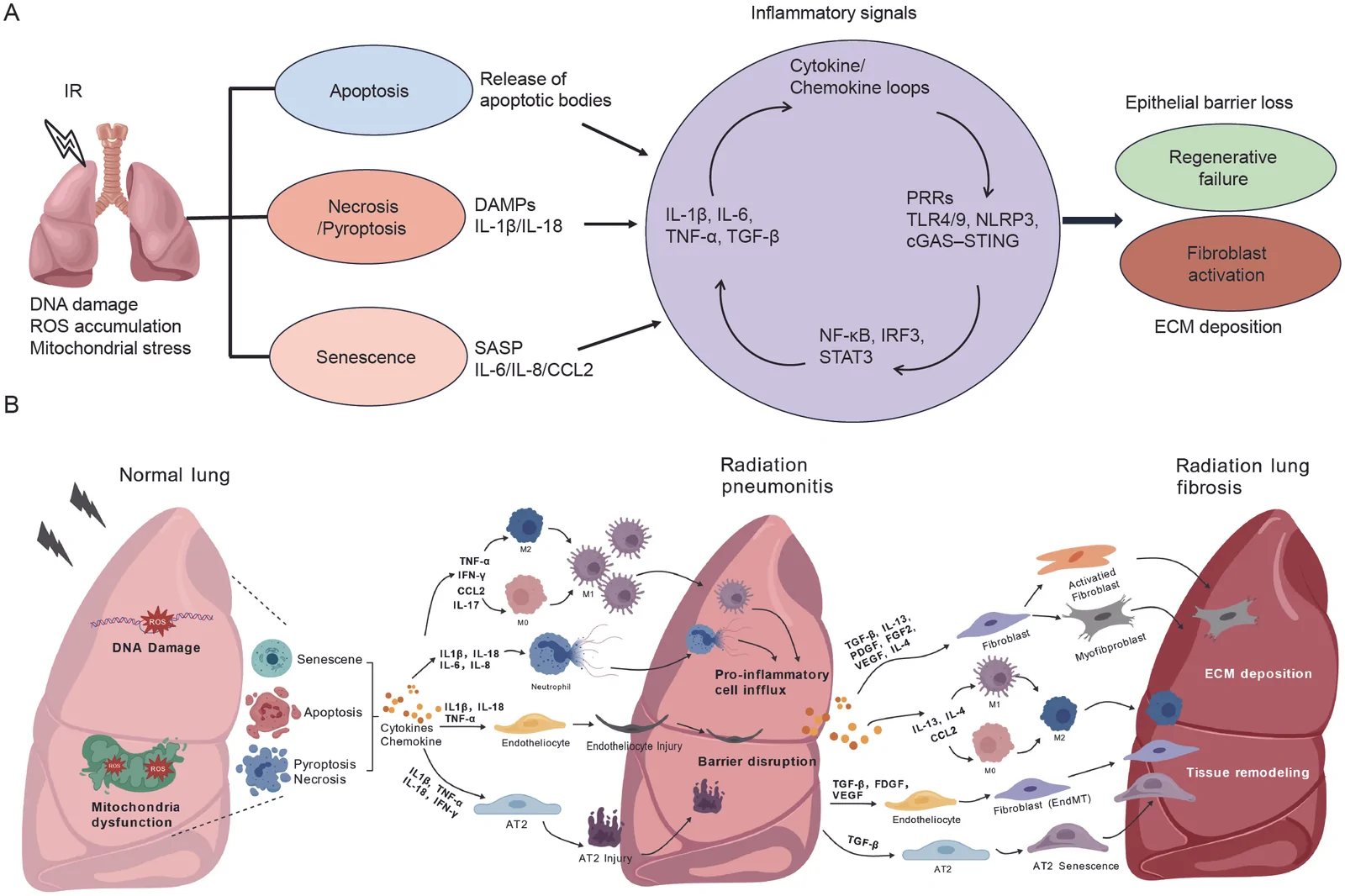
Inflammatory and fibrotic progression of RILI. **(A)** Ionizing radiation induces DNA damage, oxidative and mitochondrial stress, cell death, and senescence, activating inflammatory pathways that impair epithelial repair and promote fibroblast activation. **(B)** RILI progresses from early tissue injury and immune-cell recruitment to radiation pneumonitis and, when unresolved, radiation lung fibrosis. AT2, alveolar type 2 epithelial cell; CCL2, C–C motif chemokine ligand 2; cGAS–STING, cyclic GMP–AMP synthase–stimulator of interferon genes; ECM, extracellular matrix; EndMT, endothelial-to-mesenchymal transition; FGF2, fibroblast growth factor 2; IFN-γ, interferon gamma; IL, interleukin; IR, ionizing radiation; IRF3, interferon regulatory factor 3; M0, unpolarized macrophage; M1, classically activated macrophage; M2, alternatively activated macrophage; NF-κB, nuclear factor kappa B; NLRP3, NLR family pyrin domain-containing 3; PDGF, platelet-derived growth factor; PRRs, pattern-recognition receptors; RILI, radiation-induced lung injury; ROS, reactive oxygen species; SASP, senescence-associated secretory phenotype; STAT3, signal transducer and activator of transcription 3; TGF-β, transforming growth factor beta; TLR4/9, Toll-like receptors 4 and 9; TNF-α, tumor necrosis factor alpha; VEGF, vascular endothelial growth factor.

RILI is best viewed as a dynamic pathological continuum rather than a discrete inflammatory toxicity. It is initiated by radiation-induced epithelial and endothelial injury and progresses through persistent inflammation, defective repair, and ultimately fibrotic remodeling (8). Following irradiation, alveolar epithelial cells and pulmonary microvascular endothelial cells are among the earliest and most functionally important targets, undergoing oxidative stress, DNA damage, mitochondrial dysfunction, and disruption of the alveolar-capillary barrier (9). These early events trigger the release of damage-associated molecular patterns (DAMPs) and inflammatory mediators, promoting immune cell recruitment into irradiated lung tissue. The subsequent course of RILI is shaped by radiation-induced alterations in cellular fate, including apoptosis, pyroptosis, necroptosis, and senescence (10, 11). Together, these processes drive epithelial and endothelial cell loss, amplify inflammatory signaling, sustain maladaptive repair, and ultimately promote fibroblast activation, extracellular matrix deposition, and chronic fibrotic remodeling (**Figure 1**).

### 3.2 Systemic anticancer therapies as modifiers of RILI risk

In combined-modality treatment, systemic therapy complicates interpretation of the RILI continuum because pulmonary events may reflect intrinsic drug toxicity, modification of radiation injury, or both. Agents differ substantially in these effects. Some agents mainly introduce intrinsic pneumonitis or ILD risk (12), whereas others may plausibly modify radiation injury through radiosensitization, impaired repair, vascular injury, or immune activation (13). Consequently, post-radiotherapy pulmonary toxicity may be radiation-dominant, drug-dominant, or interaction-modified (14). Accordingly, the effects of systemic therapy on RILI risk should be considered in terms of direct pulmonary toxicity and biological amplification of RILI (**Table 1**). To standardize the terminology used throughout this review, pulmonary events after radiotherapy and systemic anticancer therapy are grouped into four operational attribution phenotypes (**Box 1**). These categories are probabilistic rather than formally validated diagnostic entities and should be reassessed as clinical and imaging findings evolve.

**Table 1:** Representative systemic therapies relevant to post-radiotherapy lung injury risk and causal attribution.

Category	Representative agent	Predominant risk pattern	Key evidence and attribution
Cytotoxic chemotherapy	Gemcitabine	Direct pulmonary toxicity and possible biological amplification	Associated with pulmonary toxicity during thoracic chemoradiotherapy; RAD51 inhibition provides a plausible mechanism for amplified radiation injury (15, 16).
	Taxanes (paclitaxel and docetaxel)	Clinically consistent with biological amplification, but not definitive	Clinical studies link taxane-based regimens to increased radiation pneumonitis and pulmonary metabolic response, although additive toxicity cannot be excluded (17, 18).
	Concurrent platinum doublets	Regimen-dependent interaction	Risk varies with the partner drug and lung dose; paclitaxel–carboplatin produced more grade ≥2 radiation pneumonitis than cisplatin–etoposide in a randomized trial (18).
	Pemetrexed, vinorelbine, and etoposide	Variable direct pneumotoxicity	Evidence ranges from post-marketing ILD with pemetrexed to case-level or regimen-confounded reports for vinorelbine and etoposide; RILI amplification is unproven (19–22).
Targeted therapies and antibody-drug conjugates	EGFR-TKIs (erlotinib, gefitinib, and osimertinib)	Direct pneumotoxicity; possible RT interaction	EGFR-TKIs carry intrinsic ILD risk, and pneumonitis occurs with thoracic RT, particularly with concurrent treatment; impaired epithelial repair remains mechanistically plausible (23–25).
	ALK inhibitors and selected ROS1 inhibitors	Predominantly direct pneumotoxicity	Drug-related pneumonitis is uncommon but recognized; RT-specific interaction data are insufficient, mainly complicating post-RT attribution (26, 27).
	Anti-angiogenic therapy, especially bevacizumab	Vascular and structural injury	Bevacizumab is associated chiefly with hemorrhage, impaired healing, and fistula in irradiated thoracic tissues rather than classic inflammatory pneumonitis (28–30).
	Antibody–drug conjugates, especially trastuzumab deruxtecan	Predominantly direct pulmonary toxicity	Trastuzumab deruxtecan has a clinically relevant intrinsic ILD/pneumonitis risk; prior thoracic RT may confound attribution but is not a proven RILI amplifier (31).
Immunotherapy	Post-CRT consolidation ICIs, especially durvalumab	Direct immune pneumonitis plus possible interaction with radiation-primed lung	Post-CRT durvalumab is associated with mostly low-grade pneumonitis/RP, reflecting overlap between intrinsic ICI toxicity and radiation-primed inflammation (32–34).
	Concurrent ICIs with chemoradiotherapy	Possible biological amplification; trial-design dependent	Severe pneumonitis rates varied across trials (KEYNOTE-799: 8.0% and 6.9%; PACIFIC-2: no overall excess), indicating a non-uniform signal (35, 36).
	Induction or prior ICI exposure before RT	Possible baseline susceptibility modification	Evidence is observational; dosimetry remains important, with mean lung dose the strongest predictor in a multi-institutional cohort (37, 38).

Box 1 **Operational definitions of pulmonary injury phenotypes after thoracic radiotherapy and systemic anticancer therapy.**

**Table:** 

Phenotype	Timing and systemic therapy exposure	Spatial and dose-map pattern	Attribution
Radiation-dominant RILI	Weeks to months after RT for pneumonitis; later for fibrosis. No compelling systemic-therapy link.	Field-centered, greatest in moderate- or high-dose lung; strong dose-map concordance.	Classical RILI without a more plausible alternative cause.
Interaction-modified RILI	During or soon after concurrent or sequential therapy; onset, severity, or course is atypical for RT alone.	Radiation-centered or dose-related, with possible limited out-of-field extension.	RILI plausibly modified by systemic therapy.
Drug-dominant pneumonitis	During or after recent exposure to an ILD-risk agent; timing may not fit the expected RILI window.	Bilateral, multifocal, migratory, or diffuse; weak or absent dose-map concordance.	Intrinsic drug toxicity is the leading cause.
Non-radiation mimic	Timing is not aligned with RT or systemic therapy and favors another disorder.	Dose-discordant; distribution follows the alternative diagnosis.	Infection, progression, embolism, hemorrhage, or cardiac dysfunction is more plausible.

### 3.3 Preclinical mechanisms of radiation–systemic therapy interactions

Preclinical studies help determine whether observed clinical overlap reflects a biologically plausible interaction, although the evidence remains uneven across treatment classes. Early chemotherapy experiments showed that normal-lung radiosensitization may depend on drug timing, but their translational relevance is limited by older single-dose irradiation models (39, 40). Targeted-therapy data are sparse and inconsistent: gefitinib may intensify early inflammation without increasing late fibrosis, whereas olaparib did not exacerbate lung injury after limited-field irradiation (41–43). Evidence is more extensive for PD-1 blockade. Most, but not all, models report greater pulmonary injury when ICIs are combined with thoracic irradiation, involving pulmonary T-cell recruitment, IL-17A-dependent neutrophilic inflammation, profibrotic signaling, and RGMb-mediated p38 MAPK/ERK activation (44, 45). Sequence-controlled studies further suggest that treatment during or shortly after irradiation may be more injurious than delayed administration (46–48). Overall, these findings support treatment-, model-, and sequence-dependent interactions rather than a uniform synergistic effect (**Table 2**).

**Table 2:** Representative preclinical studies of radiation–systemic therapy interactions relevant to RILI.

Study and model	Treatment design	Lung outcomes and interpretation
Chemotherapy		
CBA mice (39)	Radiation: Graded single-dose thoracic irradiation Systemic therapy: Cyclophosphamide Sequence: −28 to +28 days relative to RT	Endpoints: Breathing rate; dose–response indices of lung injury Principal findings: Enhancement was timing dependent and greatest when cyclophosphamide was given before or with RT. Translational limitations: Historical model; non-fractionated RT; limited mechanistic assessment.
C57BL/6J mice (40)	Radiation: 12 Gy whole-thorax irradiation Systemic therapy: Gemcitabine, 120 mg/kg Sequence: 2 h before RT	Endpoints: Lung histology; TNF-α, IL-1α, and IL-6 Principal findings: The combination increased cytokine expression and early interstitial pneumonitis, peaking at 1 week Translational limitations: Single-dose RT; follow-up limited to 4 weeks.
Targeted therapy		
C57BL/6 mice (41)	Radiation: 12 Gy whole-thorax irradiation Systemic therapy: Gefitinib, 20–200 mg/kg/day Sequence: Days 0–5 or 14–19 after RT	Endpoints: Fibrosis score and lung collagen at 5 months Principal findings: Early treatment did not worsen fibrosis; delayed treatment at 200 mg/kg reduced fibrosis and collagen. Translational limitations: Non-tumor-bearing model; highest dose greatly exceeded clinical exposure.
Wistar rats (42)	Radiation: 20 Gy thoracic irradiation Systemic therapy: Gefitinib, 50 mg/kg/day Sequence: Days 1–14 after RT	Endpoints: Inflammation (day 15); fibroblasts and collagen (day 57) Principal findings: Gefitinib aggravated early pneumonitis but attenuated later fibrotic remodeling. Translational limitations: High single dose; phase-dependent effects complicate clinical extrapolation.
Urethane-induced lung tumors in A/J mice (43)	Radiation: Left hemithorax: 5 Gy × 5f every 3 days or 13 Gy × 1f; whole-thorax tolerability cohort: 13 Gy × 1f Systemic therapy: Olaparib, 50–100 mg/kg Sequence: 1 h before RT; whole-thorax cohort also 2 h after RT	Endpoints: Tumor burden; lung histology/fibrosis; weight and esophageal toxicity Principal findings: Hemithoracic combinations improved tumor response without added lung injury; whole-thorax treatment increased esophageal/systemic toxicity. Translational limitations: Heterogeneous schedules and marked field-size/adjacent-organ effects.
Immunotherapy		
C57BL/6 mice (49)	Radiation: 20 Gy whole-thorax irradiation Systemic therapy: Anti-PD-1: 200 µg before RT; 100 µg boosters Sequence: Days −4, −2, and 0; boosters on days 3, 7, 10, 14, and 17	Endpoints: 21-day survival; pulmonary and cardiac T-cell infiltration. Principal findings: The combination reduced survival and increased thoracic T-cell infiltration. Translational limitations: High single dose, short follow-up, and cardiac co-irradiation preclude lung-specific attribution.
CBA mice (48)	Radiation: 13.5 Gy whole-thorax irradiation Systemic therapy: Anti-PD-1–3 mg/kg i.p. every 3 days × 4 Sequence: Concurrent with RT	Endpoints: Survival, lung weight, and histopathology through 6 months Principal findings: Anti-PD-1 did not significantly increase RILI or fibrosis over RT alone. Translational limitations: A single strain/dose and low event rate limited power.
C57BL/6 mice (44)	Radiation: 8 Gy × 3f whole-thorax irradiation Systemic therapy: Anti-PD-1–10 mg/kg i.p. Sequence: From day −1, twice weekly for 3 weeks	Endpoints: Histology; lung immune cells; IL-17A; neutrophils Principal findings: PD-1 blockade aggravated acute RILI through γδ T-cell-derived IL-17A and neutrophilic inflammation. Translational limitations: Non-tumor-bearing, single-strain model; 28-day follow-up.
C57BL/6 mice (46)	Radiation: 15 Gy whole-thorax X-ray irradiation Systemic therapy: Anti-PD-1: 200 µg before RT; 100 µg after RT Sequence: 1 week and 30 min before RT; 1, 2, and 3 weeks after RT	Endpoints: Histology, collagen/hydroxyproline, T cells, IL-6, and TGF-β1 Principal findings: The combination produced the greatest injury; a formal interaction was detected only for TGF-β1. Translational limitations: n = 5/group; single high dose; 1-month endpoint.
mouse carbon-ion model (45)	Radiation: Whole-thorax carbon-ion irradiation Systemic therapy: Anti-PD-1 antibody Sequence: Days 1, 3, and 5 after RT; RGMb-siRNA intervention from day 7	Endpoints: Histology, fibrosis, cytokines, lung TRM cells, and RGMb–p38/ERK signaling Principal findings: The combination increased inflammatory/fibrotic markers; RGMb knockdown attenuated signaling, cytokines, and TRM-cell expansion. Translational limitations: Carbon-ion-specific model without a photon comparator; uncertain generalizability to conventional RT.
C57BL/6 mice with LLC lung metastases (47)	Radiation: 18 Gy whole-thorax X-ray irradiation Systemic therapy: Anti-PD-1 antibody, 200 μg/mouse Sequence: Days 1, 3, 5 vs days 14, 16, 18 after RT	Endpoints: Histology, apoptosis, BALF cells, and cytokines Principal findings: Early post-RT dosing caused more severe lung injury than delayed treatment. Translational limitations: n = 5/group; no sham, RT-only, or anti-PD-1-only arms; 28-day follow-up.

## 4 Clinical evidence across systemic therapy classes

### 4.1 Chemotherapy and RILI

Chemotherapy remains the classical systemic partner of thoracic radiotherapy, but its effect on RILI risk is highly regimen-dependent. Among cytotoxic agents, gemcitabine has the clearest signal for clinically relevant pulmonary toxicity during thoracic chemoradiotherapy. In a phase I study of concurrent gemcitabine and thoracic radiotherapy in stage III NSCLC, grade 3 radiation pneumonitis emerged as a dose-limiting toxicity at 450 mg/m² weekly, and 300 mg/m² weekly was defined as the maximum tolerated schedule (15). In a subsequent phase II trial, Arrieta et al. halted accrual early because 6 of 19 patients (31.6%) developed grade 3–5 radiation pneumonitis, including 1 treatment-related death, after induction gemcitabine-carboplatin followed by concurrent gemcitabine-based thoracic radiotherapy (50). Gemcitabine has also been implicated in radiation recall pneumonitis (RRP) in previously irradiated lung (51). Pemetrexed has a weaker but measurable intrinsic pulmonary signal, a Japanese phase II study reported ILD in 8 of 216 patients (3.7%), including 1 drug-related death, and post-marketing surveillance identified pemetrexed-related ILD in 12 of 683 patients (1.8%) (52). By contrast, the evidence for vinorelbine and etoposide is less direct and less consistent. Vinorelbine-associated interstitial pneumonitis is supported mainly by case reports and small series, while etoposide has been linked to pneumonitis in older combination-chemotherapy reports, including 12 cases among 50 patients (24%) treated with cyclophosphamide, methotrexate, and etoposide, although the specific contribution of etoposide cannot be isolated in that regimen (22).

Beyond direct pulmonary toxicity, chemotherapy may also increase RILI by amplifying radiation-induced injury, especially when it is delivered as part of a radiosensitizing regimen. This pattern is most evident with taxane-based therapy. In a retrospective esophageal cancer cohort, McCurdy et al. found that pneumonitis symptoms increased with greater taxane exposure, from 46% in patients who received no taxanes to 62% in those treated with induction or concurrent taxanes and 74% in those exposed during both phases (17). Taxane-exposed patients also showed a higher pulmonary metabolic radiation response on FDG-PET/CT, suggesting an enhanced inflammatory response within irradiated lung tissue (17). In locally advanced NSCLC, concurrent paclitaxel-carboplatin produced a higher incidence of grade ≥2 radiation pneumonitis than cisplatin-etoposide (33.3% vs 18.9%, P = 0.036), suggesting that pulmonary risk depends not simply on the platinum backbone, but on the partner drug and concurrent treatment intensity (18). These findings indicate that taxane-containing concurrent regimens are associated with increased radiation pneumonitis risk and are biologically consistent with radiation sensitization or inflammatory amplification (18). However, because these clinical studies cannot fully separate drug-intrinsic toxicity, additive effects, and true radiation-drug interaction, the evidence should be interpreted as supportive rather than definitive.

### 4.2 Targeted therapy and RILI

Unlike cytotoxic chemotherapy, targeted agents and antibody-drug conjugates are biologically heterogeneous, and their relationship with RILI is highly drug-specific. Their contribution to post-radiotherapy pulmonary events should therefore be interpreted according to the dominant toxicity pattern: intrinsic pneumotoxicity, possible modification of radiation injury, or vascular and structural injury in previously irradiated thoracic tissues. EGFR-TKIs represent the most clinically relevant example in which intrinsic pneumotoxicity and possible modification of radiation injury may coexist. A systematic review and meta-analysis of thoracic radiotherapy combined with EGFR-TKIs reported an overall grade ≥3 treatment-related pneumonitis rate of 3.8%, with higher severe pneumonitis after concurrent treatment than after sequential treatment (23). In a retrospective series of unresectable stage III EGFR-mutant NSCLC treated with concurrent EGFR-TKI and radiotherapy, grade 3 radiation pneumonitis occurred in 6.7% of patients (24). These observations are biologically plausible, because EGFR signaling contributes to epithelial maintenance and repair, and EGFR inhibition may interfere with recovery of injured airway or alveolar epithelium (53). At the same time, EGFR-TKIs have an intrinsic pulmonary toxicity signal even without radiotherapy. In FLAURA, ILD occurred in 4% of patients treated with osimertinib, and a Japanese real-world cohort reported grade ≥3 drug-related pneumonitis in 21 of 452 patients receiving first-line Osimertinib (54, 55). Thus, EGFR-TKI-associated pulmonary toxicity after thoracic radiotherapy should be interpreted as an overlap between drug-intrinsic ILD risk and impaired repair of irradiated lung, rather than as purely radiation-related or purely drug-related.

ALK inhibitors are better framed as agents with intrinsic pneumonitis risk rather than established amplifiers of RILI. A systematic review and meta-analysis of ALK inhibitor trials reported a pooled pneumonitis incidence of 2.14% for all grades, 1.33% for grade ≥3 events, and 0.22% for grade 5 events, with higher rates in Japanese cohorts (26). An updated meta-analysis reported a similar overall incidence of 2.92% and again noted heterogeneity among different ALK inhibitors and patient populations (27). Because thoracic radiotherapy-specific evidence remains limited, ALK inhibitors should mainly be considered drugs that complicate attribution after radiotherapy, not proven biological modifiers of RILI.

T-DXd represents a different pattern, in which direct drug-related ILD or pneumonitis dominates the clinical concern. In a pooled analysis of nine T-DXd monotherapy studies including 1,150 patients, adjudicated drug-related ILD or pneumonitis occurred in 15.4% of patients. Most events were grade 1 or 2, but grade 5 events occurred in 2.2%. The median time to onset was 5.4 months, and most first events occurred within the first year (31). These data indicate a clinically meaningful intrinsic ILD risk even without thoracic radiotherapy. In patients with prior lung irradiation, the main task is therefore to distinguish drug-dominant ILD from radiation-primed or interaction-modified lung injury, rather than to assume that T-DXd directly amplifies RILI.

Anti-angiogenic therapy, particularly bevacizumab, should be treated separately from classic pneumonitis-inducing agents. Its dominant concern is vascular fragility and impaired repair of previously injured thoracic tissues rather than inflammatory pneumonitis. Severe or fatal pulmonary hemorrhage has been reported with bevacizumab in NSCLC, especially in higher-risk settings. Earlier clinical experience showed a marked hemorrhage signal in squamous NSCLC, and a Japanese nested case-control study of 6,774 bevacizumab-treated patients found grade ≥3 hemoptysis in 23 patients, of whom 14 died (28, 29). Prior thoracic radiotherapy may further increase concern for structural complications, as case reports and small series have described tracheoesophageal fistula or related thoracic fistulas after bevacizumab exposure in previously irradiated patients (30). Bevacizumab should therefore be viewed not as a typical RILI-pneumonitis modifier, but as a potential amplifier of vascular and structural injury in irradiated thoracic tissues.

### 4.3 Immunotherapy and RILI

ICIs should be interpreted through two overlapping pathways: intrinsic immune-mediated pneumonitis and possible modification of RILI. Even without radiotherapy, ICIs carry a measurable pneumonitis risk. In a meta-analysis of 19 NSCLC trials, PD-1 inhibitors were associated with higher rates of any-grade pneumonitis than PD-L1 inhibitors (3.6% vs. 1.3%) and grade 3–4 pneumonitis (1.1% vs. 0.4%), treatment-naïve patients also had a higher rate of any-grade pneumonitis than previously treated patients (4.3% vs. 2.8%) (32). These data establish drug-intrinsic pneumonitis as a real toxicity of this class.

In the thoracic radiotherapy setting, this intrinsic risk overlaps with radiation-related inflammation and repair failure, making attribution particularly difficult. Pneumonitis after ICI-based combined treatment may reflect immune-mediated pneumonitis, conventional RILI, or an interaction between checkpoint blockade and a radiation-primed lung. This overlap is especially relevant after chemoradiotherapy or during concurrent immunoradiotherapy, where clinical studies have reported variable pneumonitis signals depending on treatment timing, trial design, lung dose exposure, and patient selection. Therefore, ICI-associated pulmonary toxicity after thoracic radiotherapy should not be described as a uniform increase in RILI risk, but as a context-dependent spectrum. Mechanistically, interaction between ICIs and RILI is biologically plausible. Radiation-induced DNA damage and cell stress can activate innate immune sensing pathways, including cGAS-STING and type I interferon signaling, while also increasing antigen release and local inflammatory signaling (56, 57). Checkpoint blockade may sustain immune activation in this radiation-primed microenvironment, lowering the threshold for clinically apparent pneumonitis in susceptible patients. The pulmonary impact of ICIs should therefore be judged according to intrinsic pneumonitis risk, treatment sequence, lung dose exposure, irradiated volume, baseline pulmonary vulnerability, and whether residual radiation injury is still biologically active at the time of ICI exposure.

## 5 Treatment sequencing as an important determinant of RILI

**Figure 2:**
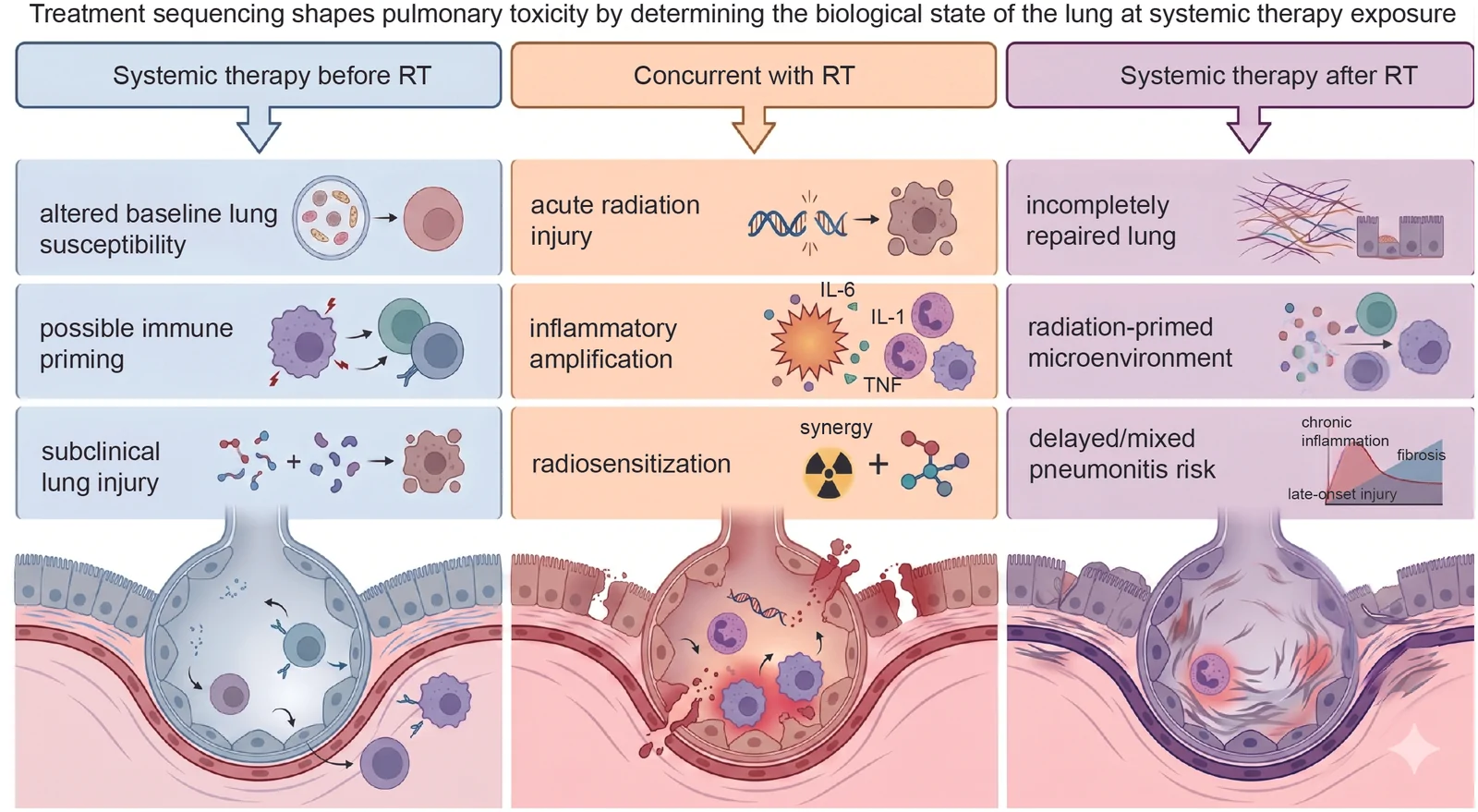
Sequence-dependent effects of systemic therapy on pulmonary toxicity. Systemic therapy before RT may increase baseline lung susceptibility; concurrent treatment may amplify acute injury through radiosensitization and inflammation; and post-RT therapy may act on incompletely repaired, radiation-primed lung, promoting delayed, mixed, or prolonged pneumonitis. These effects support sequence-specific risk assessment and monitoring.

The pulmonary consequence of a systemic agent depends not only on the drug itself, but also on the biological state of the lung at the time of exposure (**Figure 2**). This concept is supported by studies of PD-1 blockade given at different intervals after thoracic irradiation. In the study by Tong et al., mice receiving anti-PD-1 on days 1, 3, and 5 after thoracic irradiation developed more severe lung injury than those treated on days 14, 16, and 18, with pathological injury scores of 3.2 ± 0.2 and 2.2 ± 0.3, respectively (37). In the clinical cohort from the same study, grade ≥2 pneumonitis occurred in 50.0% of patients in the concurrent group and 10.6% in the sequential group (37). A separate mouse study by Yan et al. reached a similar conclusion: immediate anti-PD-1 administration after thoracic radiotherapy caused more severe lung tissue injury than delayed administration, with greater inflammatory-cell accumulation in bronchoalveolar lavage fluid and higher cytokine levels (47). Although these studies do not fully define the mechanisms of injury in humans, they support the view that treatment sequence is not merely a scheduling issue, but may influence how systemic therapy interacts with the evolving RILI continuum.

In the induction setting, the key concern is that systemic therapy may alter pulmonary susceptibility before thoracic radiotherapy begins. This is most relevant for prior ICI exposure. Chen et al. reported that thoracic radiotherapy delivered after PD-(L)1 inhibitor treatment was associated with increased incidence and severity of treatment-related pneumonitis in patients with lung cancer (58). Bi et al. further showed in a multi-institutional cohort of patients receiving thoracic radiotherapy after prior ICI exposure that mean lung dose remained the most important dosimetric predictor of radiation pneumonitis, including grade≥3 events (38). These data suggest that conventional dose-volume factors remain important, but they may operate in a clinical context already shaped by previous immunotherapy exposure. In practice, induction immunotherapy, and targeted therapy with known ILD liability, should prompt careful reassessment of respiratory symptoms, baseline imaging, and lung dose constraints before thoracic radiotherapy.

Concurrent treatment represents the setting with the most direct biological overlap, because systemic therapy is delivered during active radiation-induced tissue injury. In KEYNOTE-799, pembrolizumab given with concurrent chemoradiotherapy for unresectable stage III NSCLC resulted in grade ≥3 pneumonitis in 8.0% of patients in cohort A and 6.9% in cohort B (35). In the NICOLAS trial, early safety analysis found no grade ≥3 pneumonitis among the first 21 patients during the planned early monitoring window, and later reporting from the full cohort showed grade ≥3 pneumonitis in approximately 6.5% of patients (59, 60). These rates do not establish the dominant mechanism of injury, but they are consistent with the clinical concern that concurrent checkpoint blockade can make radiation-related lung injury more likely to become clinically significant. PACIFIC-2 further illustrates this uncertainty: simultaneous durvalumab with platinum-based CRT did not increase pneumonitis or radiation pneumonitis compared with placebo (28.8% vs. 28.7%; grade ≥3, 4.6% vs. 5.6%) (61). Sequence-specific data for targeted therapy are less robust, but a 2024 meta-analysis of thoracic radiotherapy combined with EGFR-TKIs found that concurrent treatment was less tolerable than sequential treatment (23). Thus, concurrent strategies require cautious lung-dose planning and closer monitoring, especially in patients with baseline interstitial abnormalities or limited pulmonary reserve.

Systemic therapy delivered after radiotherapy enters a lung that may appear clinically stable but may remain inflamed or incompletely repaired. Consolidation durvalumab after chemoradiotherapy provides the clearest clinical example. In PACIFIC, pneumonitis or radiation pneumonitis was more frequent with durvalumab than with placebo at the any-grade level (33.9% vs. 24.8%), whereas grade 3–4 events were uncommon and only slightly different between groups (3.4% vs. 2.6%) (33). A *post hoc* analysis showed immune-mediated pneumonitis in 9.4% of durvalumab-treated patients, with grade 3–4 events in 1.9%, and most events occurred within 3 months of durvalumab initiation (62). Another PACIFIC analysis focused on symptomatic pneumonitis or radiation pneumonitis found grade ≥2 events in 19.8% of durvalumab-treated patients and 14.1% of placebo-treated patients, whereas grade ≥3 events were similar between arms (63). Real-world data are consistent with a higher burden of clinically relevant, mostly low-grade pneumonitis after durvalumab (63). In a cohort of 1,994 patients, the 2-year incidence of grade ≥2 pneumonitis was 22.1% after CRT plus durvalumab versus 13.9% after CRT alone, and durvalumab was associated with grade 2 but not grade 3–5 pneumonitis (64). Similarly, a cohort with prospectively collected toxicity data reported a higher incidence of grade ≥2 radiation pneumonitis after adjuvant durvalumab than after CRT alone (17.8% vs. 8.8%) (34). Taken together, these findings suggest that post-CRT durvalumab may increase the clinical visibility or persistence of pneumonitis in previously irradiated lung, but they do not prove that all such events are mechanistically distinct from conventional RILI.

## 6 Detection and longitudinal monitoring of RILI

In combined-modality treatment, pulmonary surveillance should be aligned with both radiotherapy and systemic therapy exposure. Early recognition of suspected RILI is challenging because respiratory symptoms, functional decline, and imaging abnormalities may arise during or after either treatment and are not specific to radiation injury. Longitudinal assessment should therefore integrate clinical evaluation, pulmonary function testing, chest CT, and the radiation dose distribution across both treatment timelines. Serial changes in DLCO, FVC, and FEV1 help quantify functional impairment, whereas CT defines the location and evolution of lung abnormalities. These measures characterize the presence, severity, and trajectory of pulmonary injury but do not establish causality (1, 65). Investigational approaches aim to identify subclinical or regional injury more objectively. Quantitative and dual-energy CT, radiomics, and dosiomics can characterize changes in lung density, perfusion, and image–dose relationships (66–68). Functional imaging based on 4DCT, PET, SPECT, MR-Linac data, or hyperpolarized gas MRI may detect regional ventilation or gas-exchange abnormalities (69–71). Molecular probes, circulating or cellular biomarkers, exhaled breath condensate, and breathomics may provide complementary biological information (72–76). However, technical variability, limited specificity, and insufficient external validation currently preclude their routine use. Clinical assessment, pulmonary function testing, CT, and dose-map correlation therefore remain the foundation of RILI detection and longitudinal monitoring.

## 7 Attribution of pulmonary events after combination anticancer therapy

Detection of pulmonary injury does not establish its cause. After thoracic radiotherapy combined with systemic therapy, attribution is challenging because radiation injury, intrinsic drug toxicity, and other pulmonary disorders may produce overlapping clinical and radiographic findings. Evaluation should therefore integrate timing relative to both treatments, spatial concordance with the radiation dose distribution, clinical course, and the strength of competing diagnoses.

### 7.1 Temporal, spatial, and clinical assessment

Classical radiation pneumonitis most often appears within the first several months after thoracic radiotherapy, whereas fibrotic injury evolves later and reflects unresolved inflammation and abnormal repair (65, 77). Systemic therapy may further blur this pattern. Consolidation immunotherapy, for example, is often initiated while irradiated lung remains inflamed or incompletely repaired, creating temporal overlap between RILI and immune-related pneumonitis (63). Targeted agents and antibody–drug conjugates with intrinsic ILD liability pose a similar problem. Timing should therefore be assessed against both the radiotherapy course and systemic treatment exposure. Spatial concordance with the dose map is a major attribution anchor. Early RILI commonly presents as ground-glass opacity or consolidation within or near the irradiated volume, whereas late injury is characterized by volume loss, traction bronchiectasis, architectural distortion, and fibrotic consolidation (65, 78). Interaction-modified RILI may show limited extension beyond the high-dose region while retaining a field-centered or dose-related distribution. In contrast, diffuse bilateral, migratory, or dose-discordant abnormalities favor drug-dominant pneumonitis or another non-radiation cause (1, 79). Symptoms mainly reflect severity rather than etiology. Gradual deterioration accompanied by field-concordant CT changes supports RILI, whereas rapid progression, prominent infectious features, or diffuse dose-discordant abnormalities should broaden the differential diagnosis. In severe or non-resolving cases, microbiological testing and bronchoscopy with bronchoalveolar lavage may help exclude infection, alveolar hemorrhage, or malignant progression but do not independently confirm RILI (1).

### 7.2 Toward a RILI-centered attribution framework

**Figure 3:**
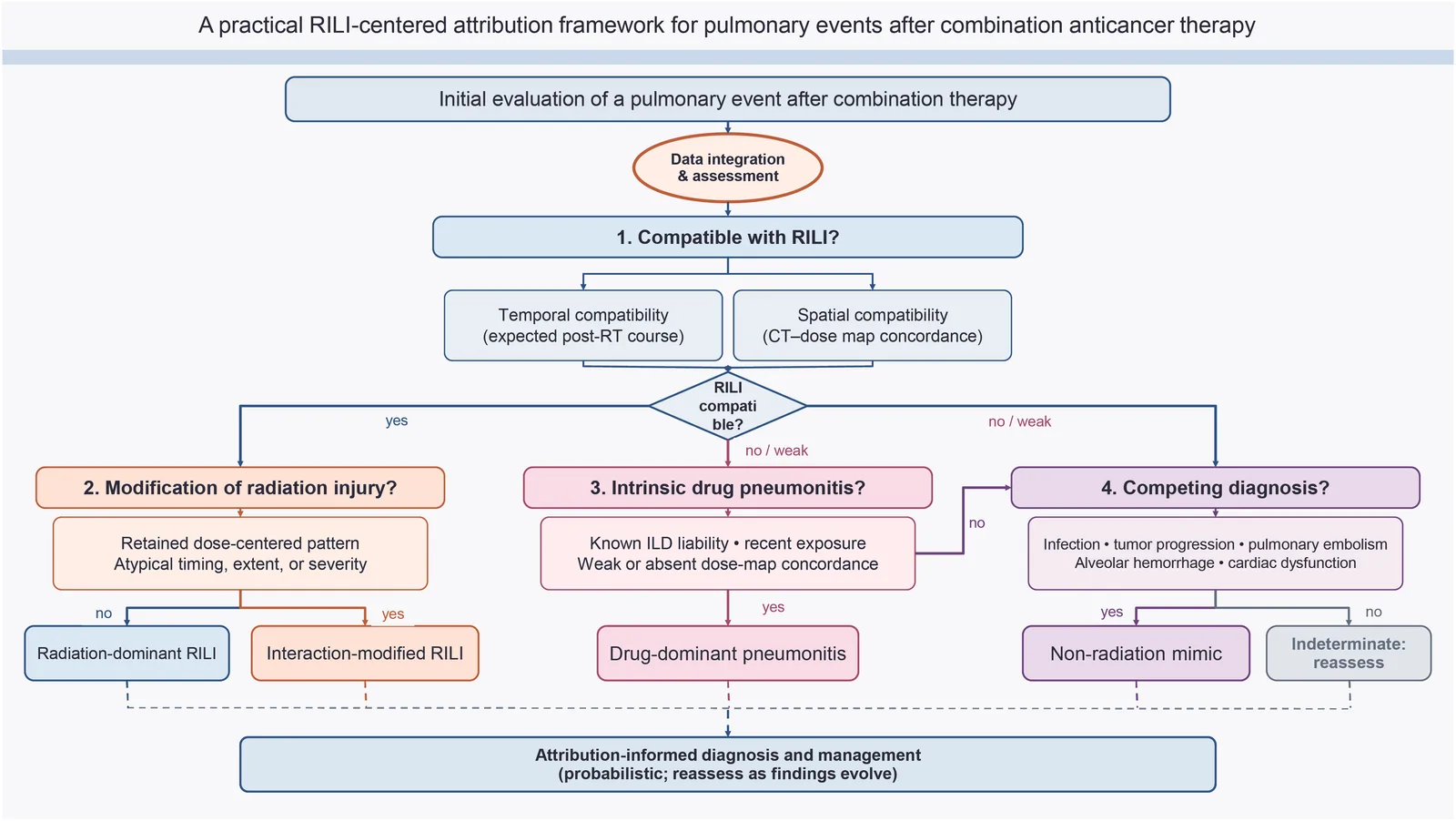
RILI-centered diagnostic framework for pulmonary events after combination anticancer therapy. Attribution integrates clinical presentation, treatment timing, CT distribution relative to radiotherapy dose maps, systemic therapy exposure, and competing diagnoses. Events are classified as radiation-dominant RILI, interaction-modified RILI, drug-dominant pneumonitis, or non-radiation mimics to guide subsequent management.

Using the four phenotypes defined in **Box 1**, attribution can be structured around three questions: whether the event is temporally and spatially compatible with radiation injury; whether systemic therapy plausibly modified its timing, extent, or severity; and whether an alternative diagnosis provides a more convincing explanation. Expected latency and strong dose-map concordance favor radiation-dominant RILI. A retained dose-centered pattern with atypical timing, extent, or severity after systemic therapy supports interaction-modified RILI. A close temporal association with a pneumotoxic agent together with a diffuse or dose-discordant pattern favors drug-dominant pneumonitis. A more compelling infectious, malignant, vascular, hemorrhagic, or cardiac explanation supports a non-radiation mimic. These assignments remain probabilistic and should be reassessed as clinical and imaging findings evolve, but they provide a practical basis for subsequent management and systemic therapy rechallenge (**Figure 3**).

## 8 Attribution-informed management of RILI

RILI management should be guided primarily by toxicity grade, with causal attribution informing the interruption and rechallenge of systemic therapy (**Table 3**). Grade 1 events require observation, symptom and oxygen-saturation monitoring, and interval imaging. Grade 2 events warrant exclusion of infectious and noninfectious mimics, temporary withholding of suspected systemic therapy when appropriate, and oral corticosteroids. Radiation-dominant pneumonitis is commonly treated with prednisone equivalent to approximately 60 mg/day for 2 weeks followed by a gradual taper (65), whereas probable ICI-related drug-dominant or interaction-modified pneumonitis generally requires prednisone 1–2 mg/kg/day tapered over at least 4–6 weeks (80). Grade 3–4 events require hospitalization, supplemental oxygen, intravenous corticosteroids, microbiological evaluation, and multidisciplinary care, with bronchoscopy when the diagnosis remains uncertain. Recurrence during tapering usually warrants re-escalation to the last effective dose and a slower taper. Failure to improve within 48-72h should prompt diagnostic reassessment and, for steroid-refractory ICI-related phenotypes, consideration of mycophenolate mofetil, intravenous immunoglobulin, or infliximab. Evidence for these agents remains limited and is not established for radiation-dominant RILI (80).

Evidence beyond corticosteroids remains limited. In a randomized phase 2 trial of 134 patients with grade 2–3 RILI, pirfenidone added to glucocorticoids improved pulmonary diffusing capacity at 24 weeks (81). A smaller randomized phase 2 trial suggested fewer pulmonary exacerbations with nintedanib plus prednisone in grade ≥2 radiation pneumonitis, but included only 30 evaluable patients and was terminated early (82). These agents were evaluated as adjuncts during symptomatic RILI rather than exclusively for established radiation fibrosis and should remain investigational. Pentoxifylline plus α-tocopherol has limited preventive evidence but no established role in treating symptomatic RILI (83). Accordingly, **Table 3** distinguishes standard grade-based management from selected emerging strategies supported by prospective clinical data.

**Table 3:** Standard management and selected emerging or investigational strategies for post-radiotherapy lung injury.

Clinical setting	Intervention	Suggested dose and regimen	Evidence and clinical role
Standard management			
Grade 1 RILI (asymptomatic radiographic changes)	Observation; serial assessment of symptoms, oxygen saturation, and imaging; no routine corticosteroids	Not applicable	Consensus- and guideline-supported standard (1)
Grade 2 radiation-dominant pneumonitis	Exclude mimics; oral prednisone or equivalent	Prednisone 0.5 mg/kg/day based on ideal body weight (approximately 40 mg/day) for 2 weeks, then taper over a total of 4–6 weeks	ESTRO clinical practice guideline; no randomized comparison of corticosteroid regimens (1).
Grade 2 ICI-related drug-dominant or interaction-modified pneumonitis	Withhold ICI; exclude infection and other mimics; oral prednisone or equivalent	1–2 mg/kg/day; taper over 4–6 weeks after clinical improvement	SITC guideline standard; extrapolated to interaction-modified RILI (84)
Grade 3 or 4 pneumonitis	Hospitalize; provide oxygen; stop suspected systemic therapy; give IV corticosteroids; perform microbiological testing ± bronchoscopy/BAL; obtain MDT input	1–2 mg/kg/day; transition to oral corticosteroids after improvement and taper over at least 4–6 weeks	Guideline standard for severe ICI pneumonitis; severe radiation-dominant RP consensus also supports initial intravenous corticosteroids (1, 80, 85).
Recurrent, steroid-dependent, or steroid-refractory ICI-related pneumonitis	Reassess diagnosis and infection; re-escalate corticosteroids for recurrence; consider MMF, IVIG, or infliximab if refractory	Return to the last effective steroid dose and taper more slowly. If no improvement within 48–72 h: MMF 1–1.5 g twice daily, IVIG 2 g/kg over 2–5 days, or infliximab 5 mg/kg IV once	Low-certainty ICI-pneumonitis guidance; not established for radiation-dominant RILI (80, 84)
Clinical emerging or investigational strategies			
Grade 2 or 3 RILI	Pirfenidone plus glucocorticoids	Pirfenidone 200 mg three times daily in week 1, 300 mg three times daily in week 2, and 400 mg three times daily in weeks 3–24; prednisone equivalent 40 mg/day for 2 weeks, then taper in 10-mg steps every 2 weeks over 6–8 weeks	Multicentre randomized phase 2 trial (n = 134); improved DLCO at week 24, but not yet established as standard care (81).
Newly diagnosed symptomatic radiation pneumonitis (grade ≥2)	Nintedanib plus prednisone taper	Nintedanib 150 mg twice daily for 12 weeks with an 8-week prednisone taper	Randomized placebo-controlled phase 2 trial (30 evaluable patients); fewer pulmonary exacerbations, but confirmation is required (82).
Prevention during thoracic radiotherapy	Pentoxifylline plus α-tocopherol	During radiotherapy: pentoxifylline 400 mg three times daily plus α-tocopherol 300 mg twice daily; for 3 months after radiotherapy: 400 mg/day plus 300 mg/day	Older single-center randomized study (n = 91); preventive signal only and not guideline-endorsed (83).

## 9 Conclusion and future perspectives

RILI in contemporary cancer care should be viewed as a context-dependent consequence of thoracic radiotherapy rather than a toxicity defined by dose alone. Its clinical expression is shaped by baseline pulmonary vulnerability, prior thoracic treatment, systemic therapy exposure, and treatment sequence, making a RILI-centered framework useful for distinguishing radiation-dominant injury, interaction-modified injury, and competing non-radiation causes. Future research should move from retrospective attribution toward prospective risk assessment through standardized definitions, integrated reporting of radiation dose distribution and systemic therapy timing, and validation of imaging, functional, and circulating biomarkers. Controlled preclinical models are also needed to separate intrinsic drug toxicity from additive or sequence-dependent interactions. Incorporating these factors into treatment planning may allow radiotherapy and systemic therapy to be combined more safely without compromising anticancer efficacy.
